# Supplementary material for: Inflammatory factors and risk of lung adenocarcinoma: a Mendelian randomization study mediated by blood metabolites
Source: Front Endocrinol (Lausanne). 2024 Aug 27;15:1446863. doi: 10.3389/fendo.2024.1446863 (PMC11384989; doi:10.3389/fendo.2024.1446863)
Supplement: Supplementary file 1 [file DataSheet1.zip › supplementary files/S2/GCST90200586/sensitivity-analysis.pdf]

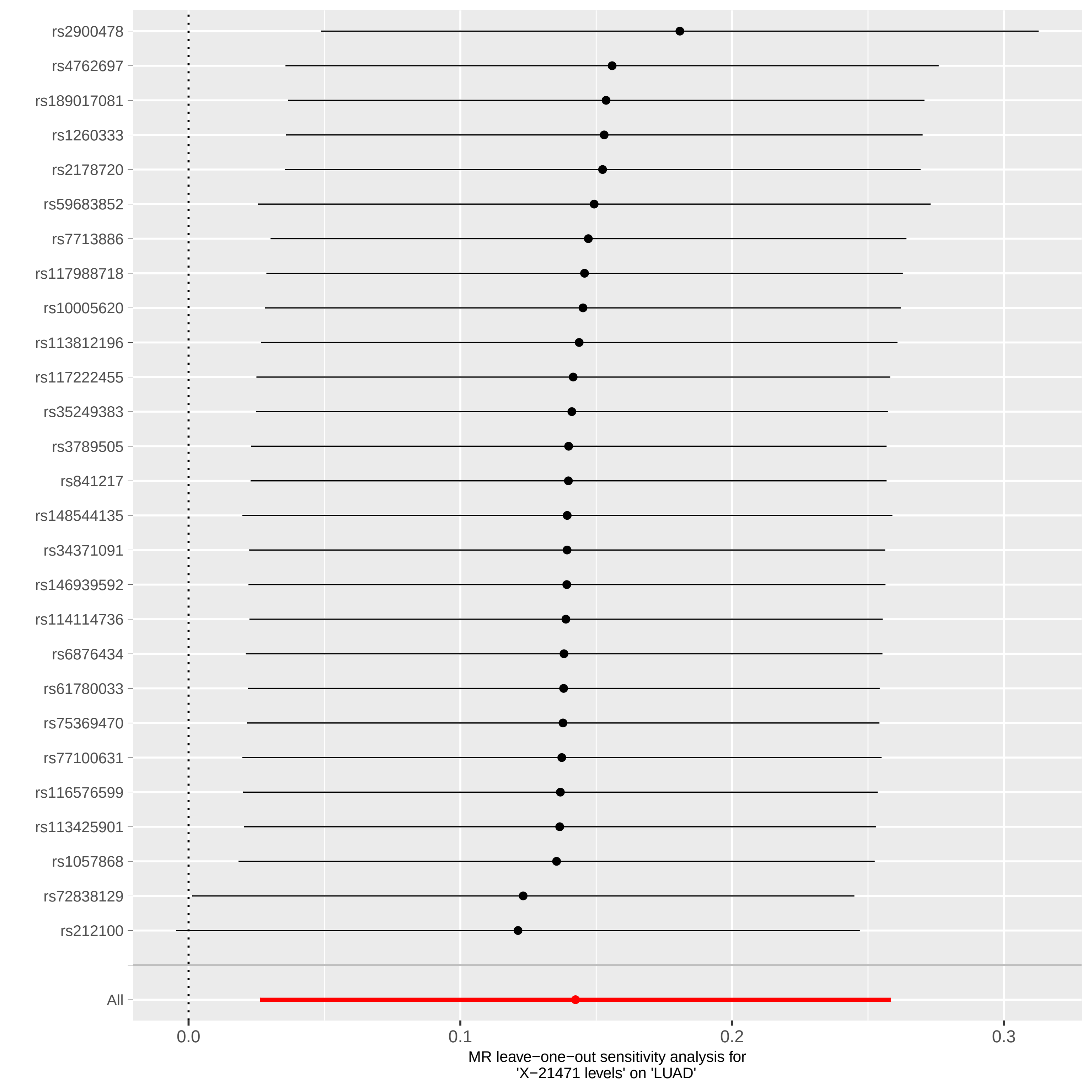

rs2900478
rs4762697
rs189017081
rs1260333
rs2178720
rs59683852
rs7713886
rs117988718
rs10005620
rs113812196
rs117222455
rs35249383
rs3789505
rs841217
rs148544135
rs34371091
rs146939592
rs114114736
rs6876434
rs61780033
rs75369470
rs77100631
rs116576599
rs113425901
rs1057868
rs72838129
rs212100
All
0.0
0.1
0.2
0.3
MR leave−one−out sensitivity analysis for
'X−21471 levels' on 'LUAD'
